# Multi-Approach Investigation Regarding the West Nile Virus Situation in Hungary, 2018

**DOI:** 10.3390/v12010123

**Published:** 2020-01-20

**Authors:** Brigitta Zana, Károly Erdélyi, Anna Nagy, Eszter Mezei, Orsolya Nagy, Mária Takács, Tamás Bakonyi, Petra Forgách, Orsolya Korbacska-Kutasi, Orsolya Fehér, Péter Malik, Krisztina Ursu, Péter Kertész, Anett Kepner, Máté Martina, Tamás Süli, Zsófia Lanszki, Gábor Endre Tóth, Anett Kuczmog, Balázs Somogyi, Ferenc Jakab, Gábor Kemenesi

**Affiliations:** 1 Virological Research Group, BSL-4 Laboratory, Szentágothai Research Centre, University of Pécs, 7624 Pécs, Hungary; brigitta.zana@gmail.com (B.Z.); lanszkizsofi@gmail.com (Z.L.); toth.gabor.endre@gmail.com (G.E.T.); kuczmog@hotmail.com (A.K.); sobalbundi@gmail.com (B.S.); jakab.ferenc@pte.hu (F.J.); 2 Institute of Biology, Faculty of Sciences, University of Pécs, 7624 Pécs, Hungary; 3 National Food Chain Safety Office, Veterinary Diagnostic Directorate, 1143 Budapest, Hungary; erdelyik@nebih.gov.hu (K.E.); MalikP@nebih.gov.hu (P.M.); UrsuK@nebih.gov.hu (K.U.); 4 National Reference Laboratory for Viral Zoonoses, National Public Health Center, 1097 Budapest, Hungary; nagy.anna@nnk.gov.hu (A.N.); nagy.orsolya@nnk.gov.hu (O.N.); takacs.maria@nnk.gov.hu (M.T.); 5 Department of Communicable Disease Epidemiology and Infection Control, National Public Health Center, 1097 Budapest, Hungary; mezei.eszter@nnk.gov.hu; 6 Department of Microbiology and Infectious Diseases, University of Veterinary Medicine, H-1078 Budapest, Hungary; Bakonyi.Tamas@univet.hu (T.B.); Forgach.Petra@univet.hu (P.F.); 7 Institute of Virology, University of Veterinary Medicine, 1210 Vienna, Austria; 8 Department of Animal Breeding, Nutrition and Laboratory Animal Science, University of Veterinary Medicine, H-1078 Budapest, Hungary; kutasi.orsolya@gmail.com (O.K.-K.); Feher.Orsolya@univet.hu (O.F.); 9 Ló-Zoo Ltd., 2162 Őrbottyán, Hungary; allatorvos@allatorvos.com; 10 PROPHYL Ltd., 18-20 Szent István Street, 7700 Mohács, Hungary; akepner@prophyl.hu (A.K.); mmartina@prophyl.hu (M.M.); tsuli@prophyl.hu (T.S.)

**Keywords:** co-circulation, West Nile encephalitis, horse, goshawk, epizootic, epidemiology, virus isolation

## Abstract

The West Nile virus is endemic in multiple European countries and responsible for several epidemics throughout the European region. Its evolution into local or even widespread epidemics is driven by multiple factors from genetic diversification of the virus to environmental conditions. The year of 2018 was characterized by an extraordinary increase in human and animal cases in the Central-Eastern European region, including Hungary. In a collaborative effort, we summarized and analyzed the genetic and serologic data of WNV infections from multiple Hungarian public health institutions, universities, and private organizations. We compared human and veterinary serologic data, along with NS5 and NS3 gene sequence data through 2018. Wild birds were excellent indicator species for WNV circulation in each year. Our efforts resulted in documenting the presence of multiple phylogenetic subclades with Balkans and Western-European progenitor sequences of WNV circulating among human and animal populations in Hungary prior to and during the 2018 epidemic. Supported by our sequence and phylogenetic data, the epidemic of 2018 was not caused by recently introduced WNV strains. Unfortunately, Hungary has no country-wide integrated surveillance system which would enable the analysis of related conditions and provide a comprehensive epidemiological picture. The One Health approach, involving multiple institutions and experts, should be implemented in order to fully understand ecological background factors driving the evolution of future epidemics.

## 1. Introduction

Along with other widely distributed flaviviruses, such as the Dengue virus or Yellow Fever virus, the West Nile virus is a major public health concern throughout multiple continents [1]. To date, nine genetic variants of the virus have been identified. However, the infectivity and neuroinvasive tendencies regarding the lineages have been largely different [2,3]. Among genetic variants, lineage 1 and lineage 2 West Nile virus (ffWNV) strains pose the largest human health risk worldwide [1]. A higher degree of infectivity and neuroinvasiveness was attributed to lineage 1 WNV until the appearance of a large number of lineage 2 WNV strains in South Africa with elevated infectivity and neuroinvasive properties [4].

The West Nile virus is transmitted by mosquitoes belonging to the *Culex* genus. However, there is growing concern in reference to the role of recently expanding invasive Aedes mosquitoes throughout Europe, establishing novel transmission scenarios [5]. The virus is maintained throughout nature in an enzootic cycle among mosquitoes and birds in which birds serve as the amplifying host while mosquitoes serve as vectors. Humans and horses are incidental or dead-end hosts which may be infected through a mosquito bite or, in very rare cases, by blood transfusion or organ transplantation [6,7]. In the majority of human cases, the infection is asymptomatic and does not bear serious consequences. In 30% of the cases, milder flu-like symptoms or encephalitic disease develops [8]. The first signs of viral illness occur within 2 to 14 days of infection. Viral infections can lead to severe neuroinvasive forms of disease (encephalitis and meningitis) in a small proportion of cases (about 1%), especially among the elderly and immunosuppressed individuals. Within these groups, mortality rates may reach up to 20% [9].

Until 2004, only the presence of lineage 1 and 3 WNV strains appeared in Europe [3]. Following the first emergence of lineage 2 WNV in Hungary in 2004 [10], this genetic variant was characterized by an explosive spread and genetic diversification in the region [11] and was responsible for several epidemics across Europe (Italy, Greece, Serbia, Austria, etc.) in both human and animal populations. The epidemics have resulted in approximately 200–300 human cases annually since the emergence of lineage 2 WNV [12,13,14,15].

According to the seasonal reports of the European Center for Disease Prevention and Control (ECDC), a significant increase in the number of human and equine cases was observed in 2018 in Hungary compared to previous transmission seasons in our region [14,15]. In this work, we presented epidemic and epizootic data regarding the 2018 WNV situation in Hungary, along with genomic data in reference to these strains and their regional progenitors.

## 2. Materials and Methods

### 2.1. Sample Collection

Samples and sample data were retrieved in a country-wide collaborative effort originating from multiple institutions. Human sample data was generously provided by the National Public Health Center, horse serologic data was obtained from the University of Veterinary Medicine Budapest, and animal-derived WNV sequences were provided by the University of Veterinary Medicine, the National Food Chain Safety Office, and PROPHYL Ltd. Mohács, Hungary.

Mosquito samples used in this study were collected as part of the regional mosquito control activities in Vojvodina, a province in Serbia, detailed by Dr. Kemenesi and colleagues [16]. Entomological surveillance of larvae and adult stages of mosquitoes was further performed in Hungary, examining the adjacent area of horse WNV cases in Dunaföldvár in 2018. We conducted a follow-up sampling for the wintering sites of mosquitoes in the ~1-km area of these horse cases in order to assess the overwintering WNV infection capacity regarding local mosquitoes (Table S2). Larvae collection was performed using the standard dipping method, while adult stages were collected via CDC light traps (BioQuip Products, CA, USA) and hand aspirators. Larvae samples and adult mosquitoes were pooled, including a maximum of 20 specimens per tube, and subjected to molecular analysis. Mosquito samples were identified via morphological characteristics [17].

A passive surveillance regarding wild bird mortality cases was performed at the Veterinary Diagnostic Directorate of the National Food Chain Safety Office. Bird carcasses examined in the avian influenza monitoring scheme were also tested for Flavivirus infection as previously described [18]. Pathology, histology, and polymerase chain reaction (PCR) were performed, and partial WNV sequences were obtained from positive cases.

### 2.2. Nucleic Acid Preparation

Total RNA was extracted using the QIAamp MinElute Virus Spin Kit (Qiagen, Hilden, Germany) in the case of mosquito homogenates and northern goshawk brain tissue samples. Total viral nucleic acid was extracted from 200 µL of human serum, ethylenediaminetetraacetic acid (EDTA)-treated whole blood, and urine samples using a High Pure Viral Nucleic Acid Kit (Roche Life Science, Mannheim, Germany).

### 2.3. PCR Amplification and Sequencing of Animal Samples

Mosquito, horse, and northern goshawk samples were subjected to WNV specific TaqMan real time reverse transcription PCR as described in [19] using the One-Step RT-PCR Kit (Qiagen, Hilden, Germany) with the following cycling conditions: Reverse transcription at 50 °C for 30 min and 95 °C for 15 min, then 40 cycles of denaturation at 95 °C for 20 s, annealing at 51 °C for 20 c, and elongation at 72 °C for 20 s. Thereafter, WNV-positive mosquito, northern goshawk, and horse samples were subjected to a nested reverse transcription-PCR (RT-PCR) system targeting the NS5 gene as described in [20] and a reverse transcription-PCR (RT-PCR) system targeting the NS3 gene (WNV_NS3_F 5’-AGCMGGAAARACRCGCAAGA-3’ and WNV_NS3_R 5’-GGTCTTTCCAACATACTCAG-3’) in order to amplify a longer genomic region regarding sequencing. PCR products were subjected to bidirectional sequencing using the BigDye Terminator v1.1 Cycle Sequencing Kit (Applied Biosystems, Foster City, CA, USA) on the ABI Prism 310 genetic analyzer platform (Applied Biosystems).

### 2.4. Molecular Diagnostics of the Human WNV Infections

Diagnostic WNV TaqMan real-time RT-PCR screening of human clinical specimens was carried out using primers and probes targeting the conserved region of the 5′-UTR and part of the capsid gene of the WNV genome. Primer and probe sequences were described previously [21]. The real-time PCR was carried out in a Light Cycler 2.0 instrument (Roche Life Science, Mannheim, Germany). The 20-µL total reaction volume contained 10 µL of template cDNA, 3.5 µL of ultra-pure DNase/RNase-free distilled water (Roche Life Science, Mannheim, Germany), 4.0 of µL ready-to-use hot start reaction mixture (Light Cycler**^®^** TaqMan**^®^** Master, Roche Life Science, Mannheim, Germany), 1.25 pmol of each primer, and 0.25 pmol of the TaqMan probe. Optimized cycling conditions were the same as those published in [21]. Confirmatory nested RT-PCR was performed using MyTaq One-Step RT-PCR Kit (Bioline, London, UK) and primer sets targeting a partial region of the NS3 coding gene of the viral genome [22]. A second-round PCR assay consisted of 2.5 µL of first-round PCR product, 7.5 µL of ultra-pure DNase/RNase-free distilled water (Invitrogen, Carlsbad, CA, USA), 12.5 µL of ready-to-use 2× My Taq Red Mix (Bioline, London, UK), and 2.0 pmol of each nested primer [22]. In full compliance to the manufacturer’s recommended guidance, 423-nucleotid-long nested RT-PCR amplicons were extracted by PCR Advanced™ PCR Clean-Up System (Viogen Biotek Corporation, New Taipei City, Taiwan). Bidirectional sequencing of the amplicons was performed using a 3500 Genetic Analyzer (Applied Biosystems, CA, USA) and the BigDye**^®^** Terminator V3.1 cycle sequencing kit (Applied Biosystems, CA, USA).

### 2.5. Serological Diagnosis of Human WNV Cases

The laboratory diagnosis regarding human WNV infections across the entire territory of Hungary was performed at the National Reference Laboratory (NRL, Budapest, Hungary) for Viral Zoonoses of the National Public Health Center (NPHC, Budapest, Hungary). Serological differential diagnostic tests for WNV, tick-borne encephalitis virus (TBEV), and Usutu virus (USUV) were performed in parallel, and all serum and CSF samples were forwarded to the NPHC’s NRL with the clinical suspicion of aseptic meningitis or viral encephalitis. Due to the milder form of the human WNV infections, commonly referred to as West Nile fever, patients afflicted with exanthema, fever, myalgia, and/or arthralgia were also investigated and involved in WNV screening during the transmission season.

First-line serological methods included the internally developed immunofluorescent assay [23] regarding the detection of WNV-, USUV-, and TBEV-specific IgG, IgM, and IgA antibodies and commercial enzyme-linked immunosorbent assays (ELISAs) for determining WNV IgM response (West Nile Virus IgM Capture DxSelect™; Focus Diagnostics, DiaSorin Molecular LLC, Cypress, CA, USA) and WNV IgG avidity (West Nile virus (WNV) avidity determination, (Euroimmun Medizinische Labordiagnostika, Lübeck, Germany). Confirmation by WNV microneutralization assay was performed in all cases. Serological cross-reactivity was excluded, and test results were evaluated according to the flavivirus vaccination status and travel history. Acute infections were defined and reported as confirmed or probable WNV infections in accordance with the EU case definition criteria.

### 2.6. In Vitro Virus Propagation

C6/36 Aedes albopictus cells (ATCC**^®^** CRL-1660™) and Vero E6 kidney cells (ATCC**^®^** CRL-1586™) were maintained in Eagle’s Minimum Essential Medium (EMEM) and Dulbecco’s Modified Eagle Medium (DMEM; Lonza, Switzerland), respectively, supplemented with 10% fetal bovine serum (Biosera, Nuaillé, France) and 1% penicillin-streptomycin (Lonza, Switzerland). C6/36 cells were maintained at 28 °C, while Vero E6 cells were maintained at 37 °C with 5% CO_2_ until reaching 70% confluency in a 24-well plate. An amount of 200 µL of supernatant from WNV PCR-positive mosquito pool homogenates, goshawk, and horse brain tissue homogenates were placed on the cell monolayer and incubated for 1 h at 37 °C. Thereafter, cells were supplemented with 1 mL of extra fresh medium and were monitored for cytopathogenic effect for seven days post-infection. After seven days, cells were frozen at −80 °C and thawed in order to lyse the cells, and 200 µL of the inoculums was used for three additional passages from the previous plates.

### 2.7. Nucleotide Sequence Analysis

Model selection in reference to accurate phylogenetic analysis was performed using the PhyML 3.0 online tool Substitution model selection section. Phylogenetic tree reconstruction was implemented using the PhyML 3.0 online tool, with the TN93+G+I substitution model performing nonparametric bootstrap analysis with 1000 replicates [24]. Trees were edited using the iTol online tool [25]. Bayesian coalescent analyses and time calibrated phylogeny were used in order to reconstruct the evolutionary dynamics of WNV. The phylogenetic tree was calibrated by attributing the sampling dates to the tips of the tree and using uncorrelated relaxed clock with lognormal distribution. Subsequently, the analyses were performed using Beast v. 1.10 software with TN93+F+G4 substitution model. The Markov chain Monte Carlo (MCMC) analysis was run for 30 million generations and sampled every 30,000 steps. The convergence assessment based on the Effective Sample Size (ESS > 200) was performed in Tracer v1.6.0. Tree Annotator program was used to summarize the trees in a maximum clade credibility (MCC) tree with a 10% burn-in. The resultant tree was visualized in FigTree v1.4.4 program.

Human-derived partial NS3 WNV sequences were compared via their identity scores. The preliminary matrix file was generated using the Sequence Demarcation Tool for Windows [26] (SDT version 1.2, www.cbio.uct.ac.za/SDT). The matrix file was edited using R software, specifically the heatmap function. Dendrogram was added with the Euclidean index and Single linkage algorithm [27].

## 3. Results and Discussion

### 3.1. Virus Detection

#### 3.1.1. PCR Screening

**Figure 1:**
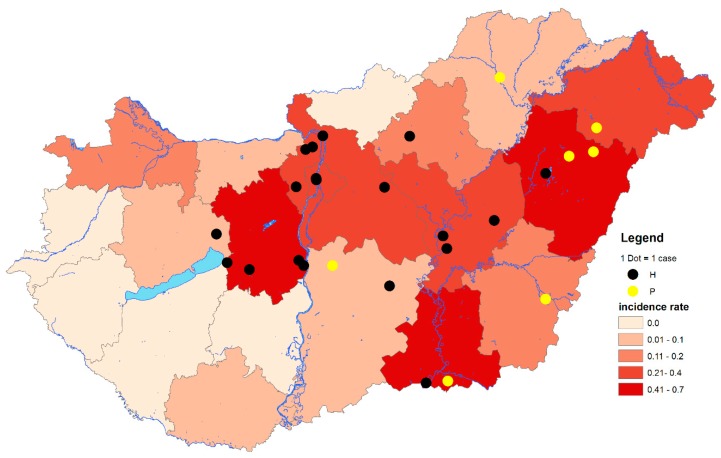
Geographical distribution of West Nile virus (WNV) polymerase chain reaction (PCR)-positive cases at the LAU 2 level and cumulative incidence rates of autochthonous and imported human WNV infections at NUTS 3 level of Hungary, from 2014 through 2017. The total number of human WNV cases between 2014 and 2017: *n* = 103. Black dots indicate PCR-positive samples containing histidine at amino acid position NS3249. Yellow dots indicate PCR-positive samples containing proline at amino acid position NS3249. LAU 2: Local Administrative Units level 2. NUTS 3: Nomenclature of territorial units for statistics level 3. IR: Incidence rates (number of WNV human infections/100,000 inhabitants).

**Figure 2:**
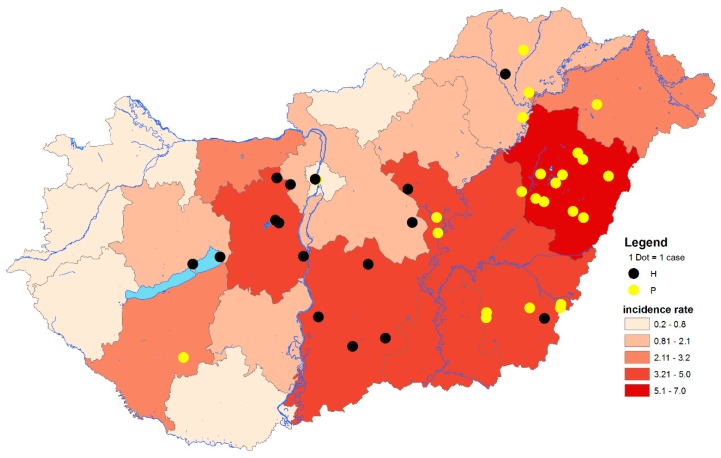
Geographical distribution of West Nile virus (WNV) PCR-positive cases at LAU 2 level and cumulative incidence rates of autochthonous human WNV infections at NUTS 3 level of Hungary, 2018. The total number of autochthonous human WNV cases in 2018: *n* = 215. Black dots indicate PCR-positive samples containing histidine at amino acid position NS3249. Yellow dots indicate PCR-positive samples containing proline at amino acid position NS3249. LAU 2: Local Administrative Units level 2. NUTS 3: Nomenclature of territorial units for statistics level 3. IR: Incidence rates (number of WNV human infections/100,000 inhabitants).

During the transmission seasons, between 2014 and 2017, *n* = 80 human patients of the *n* = 103 reported cases were examined using molecular diagnostic methods. Altogether, *n* = 32 (40.0%) were positive by both diagnostic real-time and confirmatory nested RT-PCR assays. In contrast, during the transmission season of 2018, *n* = 225 autochthonous and imported WNV cases were reported to the ECDC. In total, *n* = 53 patients were found to be positive for WNV RNA by diagnostic PCR screening, while only those samples from *n* = 46 patients (27.7%) were eventually confirmed by WNV nested RT-PCR and sequencing. Comprehensively, the results suggest a 1.4-fold increase in the number of PCR-positive cases within a single transmission period compared to the total number of cases from the previous four years (Figure 1 and Figure 2). These results are consistent with serological data, which showed a nine-fold increase in the cumulative number of human WNV cases [28].

**Figure 3:**
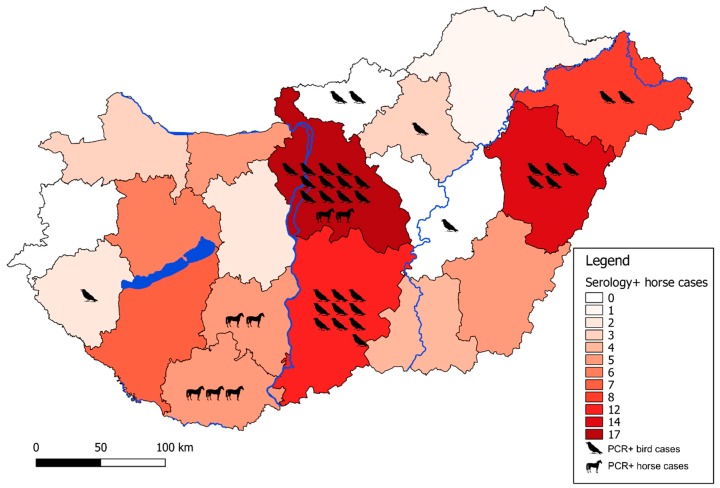
Geographical distribution of enzootic WNV cases. Color background of each county represents the number of IgM seropositive horses in that specific region from 2018 as annotated on the sidebar. Individual PCR-positive animal cases are indicated with respective pictograms to each case in that specific region.

The geographic distribution of infected birds coincided with the zones regarding PCR-positive human cases (Figure 1, Figure 2 and Figure 3). From the 150 wild and exotic bird carcasses examined from January to November 2018, we identified 14 cases of WNV related mortality. The first WNV case was detected on 2 July in a hooded crow (*Corvus cornix*) carcass from Debrecen, while the last two positive bird cases were submitted on 4 September, including a white stork (*Ciconia ciconia*) and a peregrine falcon (*Falco peregrinus*) from Tiszaalpár and Pilisvörösvár, respectively. The wide range of affected species comprised the hooded crow (3), black headed gull (*Chroicocephalus ridibundus*) (1), greenfinch (*Chloris chloris*) (1), mute swan (*Cygnus olor*) (1), monk parakeet (*Myiopsitta monachus*) (2), great tit (*Parus major*) (1), goshawk (*Accipiter gentilis*) (1), African penguin (*Spheniscus demersus*) (1), kea (*Nestor notabilis*) (1), peregrine falcon (1), and the white stork (1). As expected from our earlier experience [11,29] and regional studies [30], birds of prey were found affected, and sporadic mortality cases of hooded crows were identified at two locations. Although active monitoring of European corvid species was suggested by several studies [31,32], we only detected 6 specimens (1 magpie, 2 Eurasian jays, and 3 hooded crows) from the 130 examined corvid carcasses during the past 14 years of passive bird mortality monitoring. Nevertheless, starting an active surveillance of selected corvid species in the future may prove beneficial in the early detection of seasonal WNV circulation.

During the entomological surveillance regarding infected horse cases in Dunaföldvár, we revealed the presence of abundant larval breeding sites within close proximity to the horses (swimming pool with neglected water body and rainfall pools in artificial containers). *Culex pipiens* larvae in the swimming pool were found to be PCR-positive for WNV RNA. However, we measured an extremely low viral load, represented with PCR Ct values of 40–42, which prevented any successful *in vitro* isolation or sequencing attempts. No other mosquito species were trapped or found around the site. The total set of 36 pools from overwintering mosquitoes (*Culex pipiens*, *Anopheles maculipennis*, *Culiseta annulata*) were found to be negative for WNV RNA, which supports the previous observation regarding low-level infection rates being sufficient for the persistence of WNV in overwintering mosquito populations [33]. In addition to the role of mosquitoes, persistent bird infections should be also considered as a possible way of virus persistence and overwintering, as previously reported [34]. However, a more detailed and extensive sampling strategy likely will verify this observation in future studies. Metadata of this investigation are available as supplementary material (Table S1).

#### 3.1.2. Equine Serologic Survey

Equine cases related to WNV are reported through the Animal Disease Notification System (ADNS) of the European Commission. During the transmission season of 2017, three cases were reported which originated from Hungary [1]. In contrast to the previous year, in 2018, 91 equine cases were detected by serologic survey and virus neutralization tests. As a result, the number of cases implies an approximately 30-fold increase in WNV cases among equines.

#### 3.1.3. In Vitro Virus Propagation

Attempts to isolate the virus on C6/36 and VeroE6 cell lines proved to be successful in the case of one *Culex pipiens* mosquito pool (*n* = 50) homogenate from the Serbian/Hungarian border and one northern goshawk (*Accipiter gentilis*) brain tissue homogenate. Unfortunately, repeated attempts to isolate the virus from horse brain tissue homogenates failed regarding both cell lines. Following the third blind passage, we could not detect WNV RNA in the supernatants of lysed cells by the previously mentioned real-time RT-PCR system, likely due to the loss of viability of virus particles as a consequence regarding the condition of carcasses at the time of collection, handling, and storage methods of the samples.

### 3.2. Phylogenetic Analysis

**Figure 4:**
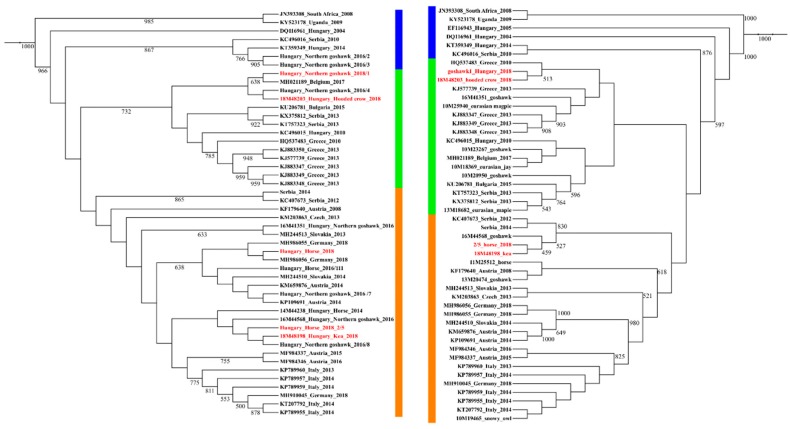
Phylogenetic representation of partial NS3 (left side) and NS5 (right side) gene sequences of Hungarian horse and wild bird samples compared to cognate sequences from the region, collected between 2010 and 2018. Progenitor African, Hungarian, and a Serbian WNV lineage 2 strains are highlighted with blue outline, while the two major subclades are indicated with green (Balkanian subclade) and orange (Central/South-West European subgroup) outline. Samples from 2018, Hungary, are highlighted with red color. Note MH021189 as an imported case from Hungary to Belgium [36].

The maximum likelihood phylogenetic analysis regarding WNV strains resulted in two bush-like groups, representing the two major subclades of WNV strains circulating simultaneously in Central Europe as previously described by others [35]. Our phylogenetic analysis revealed that the circulating WNV strains of 2018 were segregated into two different subgroups, indicating the simultaneous circulation of members from these two clades of the virus in Hungary in 2018. Partial NS3 and NS5 sequences both supported this observation. One of these phylogenetic clusters was related to members of the Balkans subclade, while the other contained WNV strains mainly from Southern and Western European countries. One Western European sequence, which was imported to Belgium from Hungary, was grouped to the Balkanian subclade. This offers a more precise evolutionary origin regarding this imported case than previously published [36] (Figure 4).

**Figure 5:**
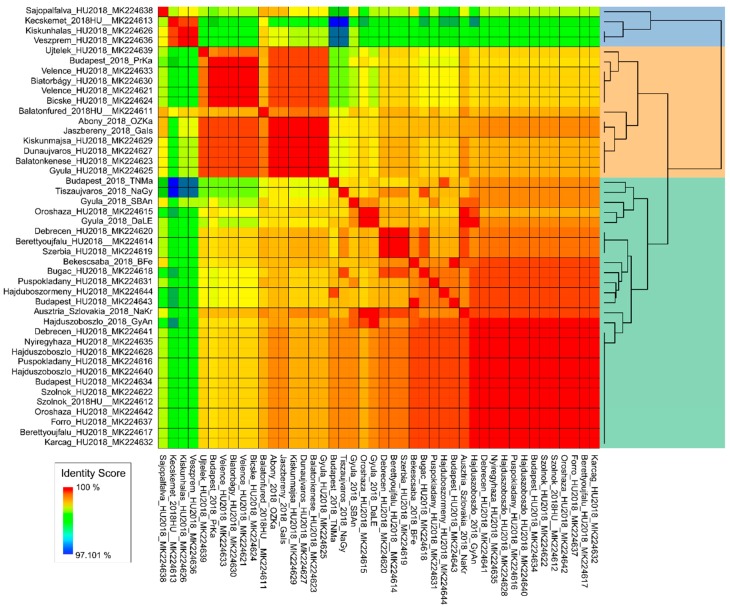
Human-derived, partial WNV NS3 sequences from the 2018 season. The figure represents similarity scores of all available human-derived WNV partial NS3 sequences from 2018. The two dominant genetic subclades are highlighted on the dendrogram with green and peach color.

The progenitor strains were segregated outside the two groups, advisably indicating the order of WNV strains emergence in time and route of introduction to Hungary. In contrast to Italy, where mostly endemic clades persist [37], we reported two WNV strains simultaneously co-circulating in Hungary. In our study, human-derived sequences permitted detailed phylogenetic analysis, mainly due to the short length of sequences, which resulted in a highly polytomic tree structure. Therefore, we indicated the presence of the two major genetic subclades on a heat map structure (Figure 5), which substantially supports the findings of the phylogenetic analysis regarding animal-derived sequences. We hypothesize the dominance of these two subclades in the affected countries in our region during the 2018 season, and quite possibly prior. However, retrospective studies may likely verify this assumption in other countries of the region as well.

**Figure 6:**
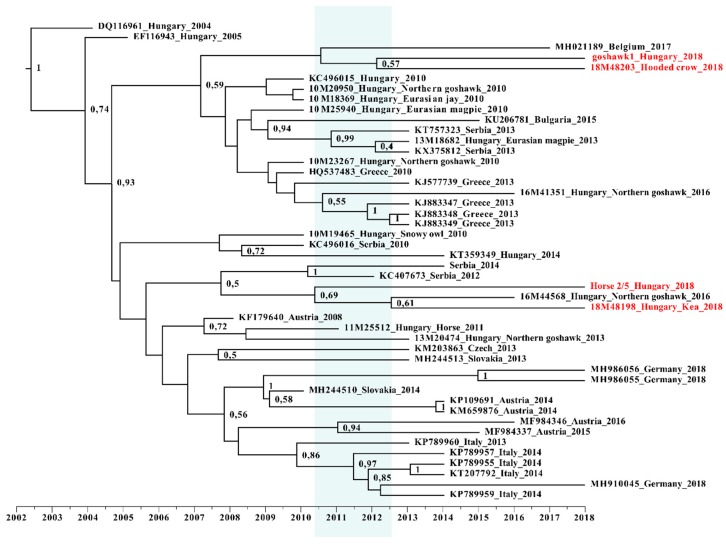
Time-calibrated Bayesian maximum clade credibility phylogenetic reconstruction of the evolution of Hungarian wild bird and horse samples. Partial NS5 gene sequences of this study, along with cognate sequences, were included in this dataset. Samples from 2018 are highlighted with red color, while the timeframe of origin is indicated with blue background.

Our results were further supported by Bayesian time-scale phylogeny (Figure 6). Strains of the 2018 outbreak most likely originated from a diversification event taking place in Hungary between 2010 and 2013, and they are represented by both subclades. Although this analysis is highly theoretical, it provides additional evidence for the absence of novel emerging strains. Our data is also confirmed by the more detailed work about the evolutionary dynamics of lineage-2 WNV [35].

## 4. Conclusions

During the transmission season of 2018, several Central-Eastern European countries reported elevated numbers of WNV infection cases among humans. In total, more than a seven-fold increase in the number of cases was observed in the EU and EU neighboring countries in 2018 compared to 2017, thus exceeding the total number of WNV cases from the past seven years [8]. Hungary was widely affected in the epidemic of 2018. To date, the exact scenario and main triggering factors are not fully understood. In a country-wide, multi-approach investigation, we aimed to understand underlying factors lurking behind the situation of 2018 in Hungary. For this purpose, we analyzed human and veterinary sequences along with country-wide human and veterinary serologic data.

Based on our sequence and phylogenetic data, the situation of 2018 was more likely caused by endemic strains rather than recently introduced novel WNV strains. We identified the presence of multiple phylogenetic subclades (Balkans subclade and the Central/South-West European subgroup) in Hungary prior to the epidemic in 2018. Therefore, we hypothesize that the main trigger factors behind the outstanding case numbers during 2018 were likely the result of favorable environmental conditions for mosquito vectors and the increased contact of these mosquitoes with native animal and human populations. However, we do not have an integrated surveillance system in the country providing a long-term comprehensive dataset to support this theory. Therefore, at this level, it is just a logical hypothesis. Since previous studies pointed out Hungary as an important ecological niche for virus diversification and dissemination in our geographic area [35], a One Health approach should be implemented in order to fully understand the ecological background factors driving annual human and veterinary cases. Our work also reflects the urgent need for a country-wide, organized surveillance system regarding the West Nile virus.

## Supplementary Materials

The following are available online at https://www.mdpi.com/1999-4915/12/1/123/s1, Table S1: Summary of overwintering mosquito pool composition. Table S2: Collection sites and their details for wintering mosquito collection in Dunaföldvár.
