# Supplementary material for: Multi-Approach Investigation Regarding the West Nile Virus Situation in Hungary, 2018
Source: Viruses. 2020 Jan 20;12(1):123. doi: 10.3390/v12010123 (PMC7019302; doi:10.3390/v12010123)
Supplement: Supplementary file 1 [file viruses-12-00123-s001.pdf]

Supplementary material

Overwintering mosquito investigation

Mosquitoes were collected with hand aspirators in 27th February, 2019. All samples were subjected for WNV screening with no positives. We further tested

Culex pipiens (*n* = 32 pool), *Culiseta annulata* (*n* = 3 pool), *Anopheles maculipennis* (*n* = 1 pool)

West Nile virus—all samples negative
Dirofilaria nematodes—all samples negative
Avian Plasmodium protozoons—all samples negative
Wolbachia bacteria—12 positive pools

Table S1. Summary of overwintering mosquito pool composition with the indication of size, site of collection and Wolbachia endosymbiont positivity.

Species (Pool)	Number of Individuals	Site	Wolbachia Positives
Culex pipiens	15	1	
Culex pipiens	20	2	
Culex pipiens	20	2	X
Culex pipiens	7	2	X
Culiseta annulata	5	2	
Culex pipiens	20	3	X
Culex pipiens	20	3	
Culex pipiens	20	3	
Culex pipiens	20	3	
Culex pipiens	14	3	
Anopheles maculipennis	1	3	
Culex pipiens	1	4	
Culex pipiens	17	5	X
Culex pipiens	20	6	X
Culex pipiens	20	6	
Culex pipiens	20	6	
Culex pipiens	20	6	
Culex pipiens	20	6	
Culex pipiens	20	6	
Culex pipiens	20	6	
Culex pipiens	20	6	
Culex pipiens	20	6	
Culex pipiens	20	6	
Culex pipiens	18	6	
Culiseta annulata	1	6	
Culex pipiens	20	7	X
Culex pipiens	20	7	X
Culex pipiens	20	7	X
Culex pipiens	20	7	X
Culex pipiens	20	7	X
Culex pipiens	20	7	X
Culex pipiens	20	7	X
Culex pipiens	20	7	
Culex pipiens	20	7	
Culex pipiens	20	7	
Culiseta annulata	3	7	

Table S2. Collection sites and their details for wintering mosquito collection in Dunaföldvár.

Site	Coordinates		Description
	N	E	
1	46°56.007	018°31.232	house basement
2	46°48.410	018°55.509	house basement
3	46°48.944	018°55.374	wine cellar
4	46°48.872	018°55.269	wine cellar
5	46°48.262	018°56.011	wine cellar
6	46°48.123	018°54.589	horse stable
7	46°48.289	018°55.356	house basement
